# Bevacizumab, With Sorafenib and Cyclophosphamide Provides Clinical Benefit for Recurrent or Refractory Osseous Sarcomas in Children and Young Adults

**DOI:** 10.3389/fonc.2022.864790

**Published:** 2022-05-25

**Authors:** Jessica Bodea, Kenneth J. Caldwell, Sara M. Federico

**Affiliations:** ^1^ Department of Oncology, St. Jude Children’s Research Hospital, Memphis, TN, United States; ^2^ Johns Hopkins All Children’s Hospital, Cancer and Blood Disorders Institute, St. Petersburg, FL, United States

**Keywords:** pediatric sarcomas, Ewing sarcoma, osteosarcoma, sorafenib, cyclophosphamide, anti-angiogeneic therapy, bevacizumab

## Abstract

**Objective:**

Children and adolescents with recurrent and metastatic solid tumors have a poor outcome. A previous phase 1 study (ANGIO1) targeting angiogenesis with bevacizumab, sorafenib, and cyclophosphamide, demonstrated a signal of activity in a subset of patients. Here we report the results of a cohort of pediatric and young adult patients treated at the recommended phase 2 doses.

**Methods:**

Electronic medical records of patients with refractory or recurrent solid tumors who received ANGIO1 therapy were reviewed. Treatment cycles lasted 21 days and included bevacizumab, sorafenib, and cyclophosphamide. Toxicities were assessed using Common Terminology Criteria for Adverse Events, v5.0. Responses were evaluated using Response Evaluation Criteria in Solid Tumors (RECIST1.1).

**Results:**

Thirty-nine patients (22 male, 17 female; median age 15 years; range 1-22 years) received the treatment regimen. The most common diagnoses included bone sarcomas (n=21; 14 Ewing sarcoma, 7 osteosarcoma) and soft tissue sarcomas (n=9; 2 rhabdomyosarcoma, 3 synovial sarcoma, 2 desmoplastic small round cell tumors, and 2 high-grade sarcoma). The most common Grade 3 non-hematologic toxicities included hypertension (2, 5.4%) and hematuria (2, 5.4%). Five patients (13.5%) had a pneumothorax (3 at progressive disease, 1 post lung biopsy, and 1 spontaneous). Common Grade 3/4 hematologic toxicities were lymphopenia (19, 51%) and leukopenia (13, 35%). Sixteen patients (43.2%) developed palmar-plantar erythrodysesthesia Grade 2 or less. A total of 297 cycles were administered. Twenty-three patients required a dose reduction of cyclophosphamide, sorafenib or bevacizumab during therapy, all of whom continued to have clinical benefit following dose modification. One patient (Ewing sarcoma) achieved a complete response after 11 cycles; 2 patients (Ewing sarcoma, high grade sarcoma) achieved a partial response following cycles 2 and 4, respectively and 20 patients had stable disease as a best response.

**Conclusions:**

Intravenous bevacizumab combined with oral sorafenib and metronomic cyclophosphamide was tolerated and required minimal supportive care or additional clinic visits. Disease stabilization for prolonged time periods was observed in greater than half of the treated patients. Patients with bone sarcoma demonstrated a signal of activity suggesting possible benefit from incorporation of the therapy as a maintenance regimen in upfront setting, or as a palliative regimen.

## Introduction

Although a multitude of therapeutic advances have improved survival rates for pediatric patients with cancer (1), there is a paucity of progress for children and adolescents with recurrent and/or metastatic solid tumors. In this patient population, outcomes remain dismal (2). Thus, new therapies targeting alternative mechanisms of action are greatly needed.

Angiogenesis is critical for oncogenesis and spread of metastatic disease. Therefore, inhibition of angiogenesis is an appealing target for patients with relapsed and refractory solid tumors. The use of anti-angiogenic drugs has become a standard practice and treatment regimen for various adult cancers, including sarcomas (3, 4). Inhibition of vascular endothelial growth factors (VEGF) and platelet-derived growth factor receptors (PDGFR) impacts angiogenesis resulting in tumor suppression and may lead to tumor response (3, 5). Further, there is significant preclinical work which has shown dual inhibition of VEGF and PDGFR produces more effective tumor suppression and increases overall survival (3).

Antiangiogenic agents have been evaluated for the treatment of pediatric malignancies (6). While combining VEGF inhibitors with other chemotherapeutics is an attractive regimen, overlapping toxicities have been dose limiting. Our institution previously evaluated anti-angiogenic agents including bevacizumab (7), a VEGF-specific recombinant, humanized monoclonal antibody which binds directly to all 4 VEGF isoforms, and sorafenib tosylate (8), a multitarget kinase inhibitor of Raf-1, BRAF, FLT-3, p39a, c-Kit, VEGFR-2, VEGFR-3, and PDGFRB. These agents were combined with metronomic low dose oral cyclophosphamide, administered daily, given the oral bioavailability and decreased systemic toxicities (9–11).

This prior phase 1 dose-escalation study (NCT00665990, ANGIO1) conducted in young adults and children with relapsed and refractory solid tumors (12) identified the recommended phase 2 doses including: bevacizumab (15mg/kg/dose IV every 21 days), sorafenib (90mg/m^2^/dose orally twice daily) and cyclophosphamide (50mg/m^2^ orally once daily). A follow-up dose expansion cohort in patients treated at the recommended phase 2 doses demonstrated that the ANGIO1 regimen was tolerated and had a signal of activity (13). Following the closure of the clinical trial, pediatric and young adult patients have been treated with this regimen at St. Jude Children’s Research Hospital. Here we report data from 39 pediatric patients, treated off study at the recommended phase 2 doses. We sought to better define the toxicities and outcomes associated to this therapeutic regimen (6–8, 12, 13).

## Methods & Materials

### Patient Population

This retrospective review was approved by the St. Jude Children’s Research Hospital Institutional Review Board. Patients with refractory or recurrent solid tumors, who were treated as per the ANGIO1 anti-angiogenic regimen were identified through pharmaceutical records. Thirty-nine electronic medical records of patients receiving the regimen between June 2009 to July 2019 were reviewed for toxicities. Two clinicians independently reviewed all anatomic and metabolic imaging to assess for response to the therapy. Patients were excluded from the analyses if they completed less than half of the first cycle of chemotherapy or did not have complete medical records or imaging available for review.

### Therapeutic Regimen

Patients received therapy, as per ANGIO1, at the recommended phase 2 doses (**Supplementary Figure 1**) (12, 13). Treatment cycle duration was 21 days and included bevacizumab (15 mg/kg, IV, day 1), sorafenib (90 mg/m^2^ PO twice daily, days 1-21), and metronomic cyclophosphamide (50mg/m^2^ PO daily, days 1-21). Patients were evaluated in their medical clinic by laboratory assessment and clinical monitoring on day 1 of each cycle and received IV bevacizumab in the outpatient setting. Oral cyclophosphamide and sorafenib were administered outpatient. All imaging obtained for disease evaluation at all time points was reviewed regardless of timing within a cycle of therapy. Timing of the initial disease response assessment varied by clinical provider (range 1-3 cycles).

### Evaluation, Response and Toxicities

Patient demographics including age, gender, disease histology, prior systemic and radiation therapy exposure were recorded. Treatment related toxicities were collected from the electronic medical record and included laboratory assessments during the duration of therapy. Toxicities were recorded using Common Terminology Criteria for Adverse Events, v5.0. Reasons for discontinuation of therapy, number of unplanned treatment related clinic visits and/or admissions, need for transfusion(s) or other significant clinical intervention were recorded. Dose adjustments, delay(s) in or holding of chemotherapy were reviewed. Disease response was independently evaluated by 2 reviewers using the Response Evaluation Criteria in Solid Tumors (RECIST 1.1) criteria for all available disease evaluations through the duration of treatment to determine best response.

## Results

### Patient Characteristics

**Table 1** summarizes patient characteristics. Thirty-nine patients (22 males, 17 females) received at least 1 cycle. Patients had a median age of 15 years (range 1-22 years). The most common histological diagnoses were bone sarcomas (n=21; 14 Ewing sarcoma, 7 osteosarcoma) and soft tissue sarcomas (n=9; 3 synovial sarcoma, 2 rhabdomyosarcoma, 2 desmoplastic small round cell tumors, 2 high-grade sarcoma). Additional diagnoses included rhabdoid tumor (n=3), hepatocellular carcinoma (n=2), Wilms tumor (n=2), clear cell meningioma (n=1), and neuroblastoma (n=1).

**Table 1:** Baseline Characteristics.

No. Patients	39
**Age on therapy (years)**	
Median (range)	15 (1-22)
**Gender, [N (%)]**	
Male	22 (56%)
Female	17 (44%)
**Histologic diagnosis**	
* Bone Sarcomas*	
Ewing Sarcoma	14 (36%)
Osteosarcoma	7 (18%)
* Other Solid Tumors*	
Rhabdoid Tumor	3 (7%)
Synovial Sarcoma	3 (7%)
Rhabdomyosarcoma	2 (5%)
Hepatocellular Carcinoma	2 (5%)
Wilms Tumor	2 (5%)
High Grade Sarcoma	2 (5%)
Desmoplastic Small Round Cell Tumors	2 (5%)
Clear Cell Meningioma	1 (3%)
Neuroblastoma	1 (3%)
**Prior therapies**	
Prior systemic regimens, [median (range)]	3 (0-6)
Prior radiotherapy, [N (%)]	28 (73.7%)
Prior lung directed radiotherapy, [N (%)]	14 (36.8%)
**Lung Disease at the start of regimen, [N (%)]**	27 (71%)

Twenty-eight patients (73.7%) had received prior radiation therapy, 14 (36.8%) of which included lung directed radiotherapy. Twenty-seven patients (71%) had lung disease at the start of the treatment regimen. Patients had received a median of 3 prior systemic therapies (range 0-6).

Two patients in the group had not received prior systemic therapy. These patients included a 20-year-old male with a sacral clear cell meningioma treated with upfront resection alone prior to receiving the therapeutic regimen and, a 15-year-old male with unresectable hepatocellular carcinoma for which other standard systemic therapy options were not available.

### Toxicities and Interventions of Interest

Toxicities related to therapy are summarized in **Table 2**. Thirty-seven (94.8%) of the 39 patients had laboratory evaluations for review. All 39 had clinical documentation for sufficient review of non-hematologic side effects.

**Table 2:** Treatment Related Toxicities and Toxicities of Interest.

Grade ≥ 3 Adverse Events	^#^N = 37 (%)
*Hematologic*	23 (62%)
Lymphopenia	19 (51%)
Leucopenia	13 (35%)
Neutropenia	7 (19%)
Thrombocytopenia	6 (16%)
*Non-Hematologic*	
Hypertension	2 (5.4%)
Emesis	1 (2.7%)
Elevated Lipase	1 (2.7%)
Weight Loss	1 (2.7%)
Transaminitis	1 (2.7%)
Hyperbilirubinemia	1 (2.7%)
**Toxicities of Interest**	
Weight Loss Grade 2	10 (27%)
Palmar-plantar erythrodysesthesia Grade 2	16 (43.2%)
Urine Protein ≥ 2+ on urine analysis	12 (32.4%)
Pneumothorax ≤ Grade 2	5 (13.5%)
Hematuria ≥ Grade 2	2 (5.4%)

^#^Two patients from cohort not included due to unreliable complete toxicity data.

The most common Grade 3/4 toxicities (n=23, 62%) were hematologic, including lymphopenia (19, 51%) and leukopenia (13, 35%). No patients experienced Grade 3/4 anemia during their treatment. Two patients (5.1%) required a platelet transfusion during the regimen, including 1 patient who was known to be platelet refractory prior to treatment initiation. One patient (2.7%) required blood transfusion for symptomatic fatigue and tachycardia. Non-hematologic toxicities greater than Grade 2 were infrequent, and included hypertension (n=2, 5.4%), nausea/vomiting, elevated lipase, weight loss, transaminitis and hyperbilirubinemia (each n=1, 2.7% respectively).

Additional treatment-related toxicities of interest included weight loss (Grade 2; n=10, 27%), palmar-plantar erythrodysesthesia (Grade 2; n=16, 43.2%), proteinuria on urine analysis of 2+ or more (n=12, 32.4%), and hematuria (n=2, 5.4%). Four patients with proteinuria and both patients with hematuria had a history of bladder involvement prior to the start of the treatment regimen. One patient with sacral clear cell meningioma had hemorrhagic cystitis resulting in removal from ANGIO1 study after 12 cycles, but then tolerated 18 cycles of therapy with the addition of oral mesna prior to developing disease progression.

Five patients (13.5%) developed a pneumothorax on therapy. Pneumothorax occurring at the time of progressive disease (n=3), following lung biopsy (n=1), and spontaneously (n=1, patient with stable disease following cycle 9). Four of the five pneumothoraces were asymptomatic Grade, and did not require intervention. One patient with pulmonary progressive disease, had a Grade 2 pneumothorax and required a chest tube placement.

Six of the 39 patients were previously treated on the phase 1 expansion cohort but were removed from the study and received ANGIO1 therapy off study. Their removal from protocol was due to the receipt of radiation therapy (n=1), development of thrombosis (n=1), weight loss (n=2), mixed response (n=1), and increased lipase (n=1). The toxicities for the 6 patients and response of cycles completed on the phase 1 study as well as off study are included in the analysis.

### Dose Modifications

Twenty-three (63.9%) patients required a dose reduction of either cyclophosphamide, sorafenib or bevacizumab during therapy. The most common reasons for dose modifications included palmar-plantar erythrodysesthesia (n=13), myelosuppression (n=8) and poor wound healing (n=7). Bevacizumab was held for upcoming surgery or radiotherapy in 3 and 2 patients respectively. Twelve patients experienced proteinuria greater than 2+ on urine analysis, and 6 of these patients experienced delay of day 1 bevacizumab while obtaining a 24-hour urine protein analysis. Formal urine protein:creatinine ratios were obtained in 4 of these patients and revealed Grade > 2 proteinuria in 3 patients. None of the patients required discontinuation of bevacizumab for this indication. Three patient’s treatment regimens included the addition of oral mesna due to a prior hemorrhagic cystitis. All patients who required a dose modification or delay in therapy continued to experience a clinical response following the dose modification.

### Tolerability

The majority of patients had zero unplanned hospital admissions (median 0, range 0-3) or greater than 1 clinic visit per 21-day cycle (median 0, range 0-7). A single patient experienced 7 unplanned clinic visits over the duration of 30 cycles of anti-angiogenic therapy, for nausea and recurrent urinary trat infections (sacral tumor and bladder involvement). A total of 297 cycles of therapy were administered to the 39 patients with a total of 8 hospitalizations and 33 unplanned clinic visits due to treatment related toxicities. The reasons for discontinuing therapy was progressive disease (n=34), enrollment on a phase I study (n=2), toxicities (n=2; fatigue, nausea and vomiting), and transfer to an outside hospital (n=1).

### Disease Response

**Figure 1:**
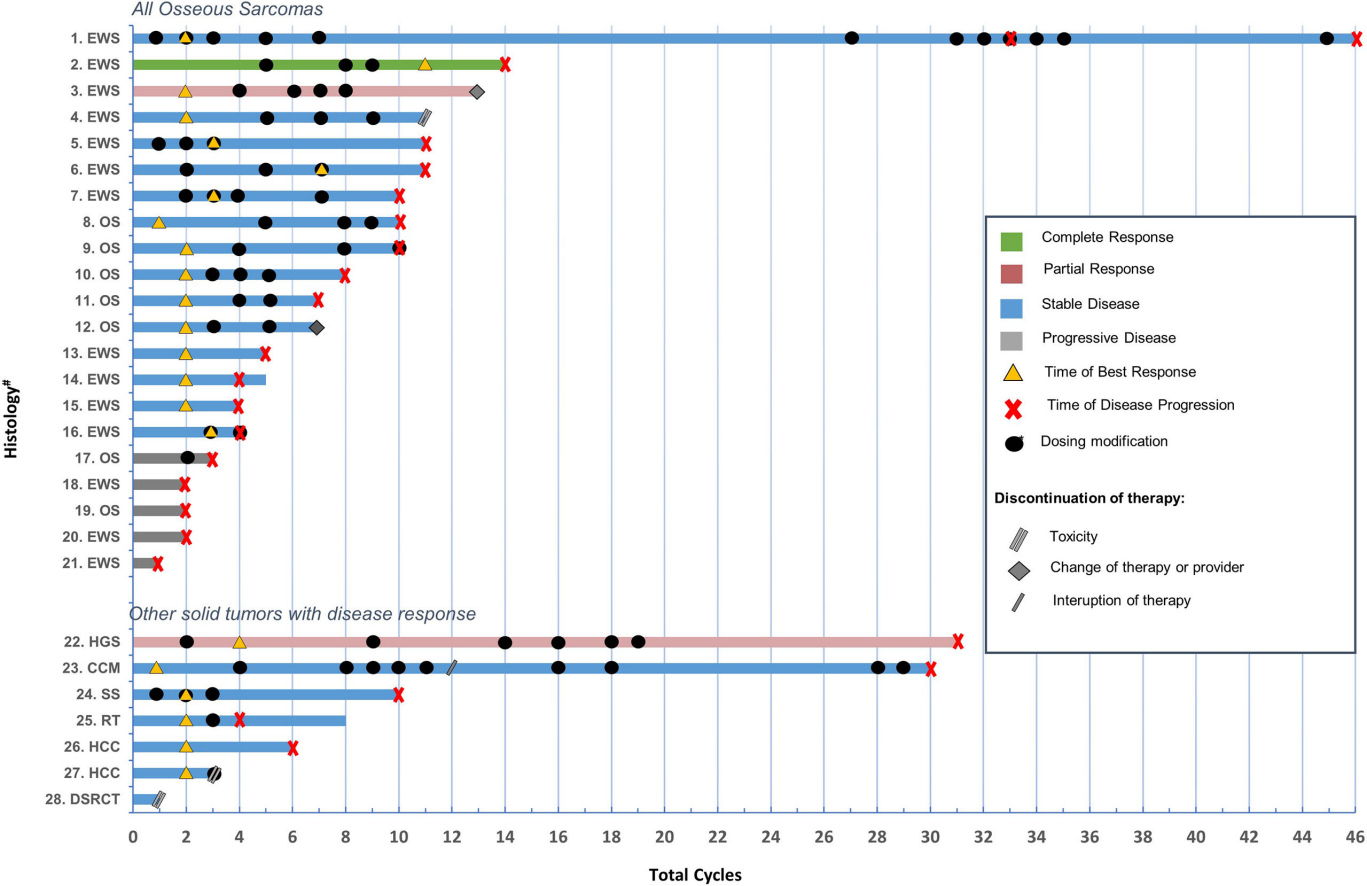
Description of treatment duartion, clinical course, timing of dosing modifications, best response, disease progression of all osseous sarcomas and additional solid tumors with response (≥SD). *Details regarding dosing modifications and unique variables is further described for each case in **Supplementary Materials**. #EWS, Ewing sarcoma; OS, osteosarcoma; HGS, high grade sarcoma; CCM, clear cell meningioma; SS, synovial sarcoma; RT, rhabdoid tumor; HCC, hepatocellular carcinoma; DSRCT, desmoplastic small round cell tumors.

The best response and treatment course for patients including timing of best response, progression, and total number of cycles treated on therapy is shown in **Table 3**. **Figure 1** demonstrates the response data of all patients with osseous sarcomas and select solid tumor diagnoses who demonstrated a clinical response greater than or equal to stable disease. Unique events including breaks in therapy, disease progression, or dose adjustments are included. Description of drug dose adjustments and rationale are described in detail in **Supplementary Table 1**.

**Table 3:** Treatment Duration and Best Response.

Treatment Course	Median (range)
*All Diagnoses*	* N=39*
Cycles to best response	2 (1-11)
Cycles to progression	4 (1-46)
Cycles on therapy	4 (1-46)
Time to death (days)	290 (35-1419)
*Bone Sarcomas*	*N=21*
Cycles to best response	2 (1-11)
Cycles to progression	6 (2-46)
Cycles on therapy	7 (1-46)
Time to death (days)	385 (97-845)
**Best Response**	N (%)
*All Diagnoses*	*N=39*
Complete Response	1 (2.6%)
Partial Response	2 (5.1%)
Stable Disease	20 (51.3%)
Progressive Disease	16 (41%)
*Bone Sarcomas*	*N=21*
Complete Response	1 (4.7%)
Partial Response	1 (4.7%)
Stable Disease	14 (66.7%)
Progressive Disease	5 (23.9%)

Twenty-three patients had a clinical response including 3 with a partial response or better. One patient, a 16-year-old with non-metastatic Ewing sarcoma of the right tibia treated with standard systemic chemotherapy and limb sparing surgery developed pulmonary recurrence a year and a half off therapy. The patient received 3 additional systemic therapies, surgery and whole-lung irradiation prior to the angiogenic treatment regimen. The patient achieved a complete response following 11 cycles of therapy and then developed disease progression after cycle 14, in the setting of poor compliance with oral cyclophosphamide and sorafenib. Two patients (Ewing sarcoma and high-grade glioma) achieved a partial response following cycles 2 and 4, respectively. Twenty patients had stable disease (median 8 cycles, range 1-46) of which 14 were bone sarcomas. Five of seven patients (71%) with osteosarcoma achieved stable disease and 11 of 14 patients (79%) with Ewing sarcoma achieved stable disease or better. The median duration of therapy for all patients was 4 cycles (range 1-46). The median duration of therapy for patients with bone tumors was 7 cycles (range 1-46). Progression occurred at a median time of 4 cycles (range 1-46) for the total cohort and at 6 cycles (range 2-46) for bone tumors. The median duration of days to death was 290 (35-1419) for all patients, and 385 (97-845) for patients with bone tumors.

Two patients are alive to date, including a patient with hepatocellular carcinoma and a patient with Ewing sarcoma. The patient with hepatocellular carcinoma received 2 cycles and discontinued therapy for surgical resection. The patient with Ewing sarcoma experienced multiple dose modifications throughout the course of treatment including delays and holding of medications for toxicities. Whenever the medications were delayed or held for periods of time, the patient would develop disease progression which would then resolve after ANGIO1 therapy resumed. The patient received a total of 46 cycles before developing disease progression while receiving the treatment regimen.

## Discussion

Angiogenesis is an important clinical target for the treatment of patients with relapsed and refractory solid tumors. This retrospective review of a cohort of 39 heavily pre-treated pediatric and young adult patients with relapsed and refractory solid tumors demonstrated that the anti-angiogenic regimen including intravenous bevacizumab and oral sorafenib combined with oral metronomic cyclophosphamide was tolerated and demonstrated clinical benefit in multiple tumors including high grade sarcoma, clear cell meningioma, synovial sarcoma, rhabdoid tumor, hepatocellular carcinoma, and desmosplastic small round cell tumor. However, particular benefit was noted in a subset of patients, most notably, those with bone tumors.

Studies evaluating anti-angiogenic therapies for the treatment of osteosarcoma and Ewing sarcoma have demonstrated variable clinical responses and need further investigation (6). Numerous agents targeting angiogenesis, including multiple tyrosine kinase inhibitors, have been evaluated for the treatment of osteosarcoma. Although bevacizumab demonstrated preclinical responses in osteosarcoma, single agent bevacizumab in the clinical setting did not increase survival (14). However, combination studies including bevacizumab have had variable results, with some studies demonstrating clinical benefit (15), and others without benefit (16, 17). Sorafenib has demonstrated activity *in vitro* and *in vivo* preclinical models of osteosarcoma with decreased tumor volume and lung metastasis following drug exposure (18). In a phase II study, single agent sorafenib led to improved progression free survival (PFS) in select patients with osteosarcoma (19, 20). In addition to sorafenib, numerous other multi-targeted tyrosine kinase inhibitors (MTKIs) have also been investigated (6). Although pazopanib demonstrated activity against osteosarcoma in preclinical studies, it failed to prevent progression in the clinical setting (21–23). Regorafenib demonstrated improved PFS in adults with osteosarcoma in two randomized control trials (24, 25) and in the CABONE study cabozantinib demonstrated a 33% longer PFS in 37% of patients with osteosarcoma (26). Numerous additional studies evaluating combinations of MTKIs with systemic chemotherapies are ongoing, yet the most effective antiangiogenic regimen have yet to be identified (6). Clinical response was demonstrated in a phase 2 trial of sorafenib and everolimus for patients with high-grade progressive osteosarcoma; however, this combination had toxic effects leading to interruptions of therapy or dose reductions in 66% of patients, highlighting the difficulty with the toxic therapeutic window (27).

While prior preclinical studies indicated that VEGF-A and PDGF were promising therapeutic targets for Ewing sarcoma, few studies evaluating antiangiogenic regimens have been conducted in this patient population. Numerous case reports and small case series have reported variable responses to therapy. Case reports using a regimen containing bevacizumab, vincristine, irinotecan, and temozolomide reported 3 patients with clinical responses including complete remission, partial response, and disease stabilization (16, 28). Another positive single case study reported prolonged remission following maintenance therapy with pazopanib in a patient with metastatic disease (29). Alternatively, other antiangiogenic regimens have produced negative responses including bevacizumab combined with gemcitabine and docetaxel (30), single agent axitinib (31) and single agent imatinib (30, 32).

Many patients experienced significant clinical benefit following administration of ANGIO1 therapy. In our treatment cohort, 16 (76.2%) of 21 patients with relapsed osseous sarcomas demonstrated a response of stable disease or better. These results are consistent with prior studies demonstrating anti-angiogenic therapeutic responses in osseous tumors (6, 7, 10). Additionally, seven patients with non-osseous disease demonstrated stable disease or better. Four of these diagnoses included synovial sarcoma, high-grade sarcoma, desmoplastic small round cell tumor (DSRCT) and rhabdoid tumor. Although these are rare cancer diagnoses, patients with relapsed synovial sarcoma have demonstrated prolonged response when treated with pazopanib as a single agent (33). Additionally, some patients with DSRCT and non-rhabdomyosarcoma soft tissue sarcomas have demonstrated partial response and disease stabilization, to anti-angiogenic therapy (34, 35). DSRCT has also been shown to demonstrate clinical response when pazopanib is used in combination with other systemic drugs such as vincristine and irinotecan (35).

The final 3 patients who demonstrated clinical benefit in the cohort included 2 with hepatocellular carcinoma (HCC) and one with a clear cell meningioma. HCC tumors are known to be hypervascular and have dysregulated angiogenic pathways (36). In the adult setting, one trial demonstrated an increased survival of 2.8 months over placebo when using single agent sorafenib in patients with advanced HCC (36, 37). Further, regorafenib has been utilized in patients with HCC who have failed or progressed while on treatment with sorafenib and has demonstrated survival benefit to those patients (38, 39).

The 39 patients who received the antiangiogenic regimen required minimal supportive care, with many patients experiencing prolonged periods of disease stabilization. Although more than half of the patient required dose reductions for toxicity, patients did not experience unexpected hospital admissions, clinic visits, or increased transfusions. While the most common toxicities experienced by patients were hematologic, importantly these patients did not require significant transfusions or admissions for febrile neutropenia. Thirty-four of 39 patients had zero hospital admissions while receiving the therapeutic regimen. Approximately half of the patients only required one clinic visit every 21 days to receive the IV bevacizumab. Furthermore, of the 19 patients that had an unplanned visit, 11 required only 1 extra clinic visit throughout the total duration of treatment.

Clinically significant non-hematologic toxicities were rare, most commonly including palmar-plantar erythrodysesthesia and pneumothorax. Those experiencing palmar-plantar erythrodysesthesiadid not have interference with their daily functioning and all had improved symptoms with dose modifications or treatment with emollients. Although pneumothorax was previously described in the phase 1 of this regimen as a common occurrence in 25% of patients (13, 40), the rate of pneumothorax in this cohort was lower at 12.8% and most frequently occurred at the time of disease progression (n=3). The significance of this is unclear due to several study limitations, including sample size, numerous cancer diagnoses and variable prior treatment regimens. Further evaluation will be necessary and would be best evaluated in a randomized trial.

There are numerous clinical applications of this treatment regimen that may benefit patients going forward. First, the clinical benefit of stable disease or better in conjunction with manageable toxicities and a decreased need of frequent medical visits makes this an appealing palliative regimen. Additionally, for patients who reside in countries with limited access to supportive care, this therapeutic treatment may be beneficial when compared to the side effect profiles of cytotoxic chemotherapeutic regimens (6). Finally, the signal of activity demonstrated in bone sarcomas suggests that it may be beneficial in if incorporated in an upfront regimen, either in combination with other cytotoxic chemotherapies or as a maintenance regimen, such as that currently done as standard of care with neuroblastoma and rhabdomyosarcoma (41, 42). Further studies with larger sample sizes and randomized controls need to be conducted for all anti-angiogenic regimens in the future to best evaluate the utility of the therapies being studied, as well as determine the best therapeutic schedule.

## Data Availability Statement

The original contributions presented in the study are included in the article/**Supplementary Material**. Further inquiries can be directed to the corresponding author.

## Ethics Statement

The studies involving human participants were reviewed and approved by the St. Jude Children’s Research Hospital Institutional Review Board. Written informed consent from the participants’ legal guardian/next of kin was not required to participate in this study in accordance with the national legislation and the institutional requirements.

## Author Contributions

JB, SF, and KC contributed to conception and of the study. JB organized the database and performed the statistical analysis. JB wrote the first draft of the manuscript. JB and SF wrote sections of the manuscript. All authors contributed to manuscript revision, read, and approved the submitted version.

## Funding

This research was supported by St. Jude Children’s Research Hospital. Supported in part by Cancer Center Grant CA23099 and Cancer Center Support CORE Grant P30 CA 21765 from the National Cancer Institute and by the American Lebanese Syrian Associated Charities.

## Conflict of Interest

The authors declare that the research was conducted in the absence of any commercial or financial relationships that could be construed as a potential conflict of interest.

## Publisher’s Note

All claims expressed in this article are solely those of the authors and do not necessarily represent those of their affiliated organizations, or those of the publisher, the editors and the reviewers. Any product that may be evaluated in this article, or claim that may be made by its manufacturer, is not guaranteed or endorsed by the publisher.
