# Supplementary material for: Bevacizumab, With Sorafenib and Cyclophosphamide Provides Clinical Benefit for Recurrent or Refractory Osseous Sarcomas in Children and Young Adults
Source: Front Oncol. 2022 May 25;12:864790. doi: 10.3389/fonc.2022.864790 (PMC9174993; doi:10.3389/fonc.2022.864790)
Supplement: Supplementary file 1 [file DataSheet_1.docx]

Supplementary Material

# Supplementary Figures and Tables

## Supplementary Figures


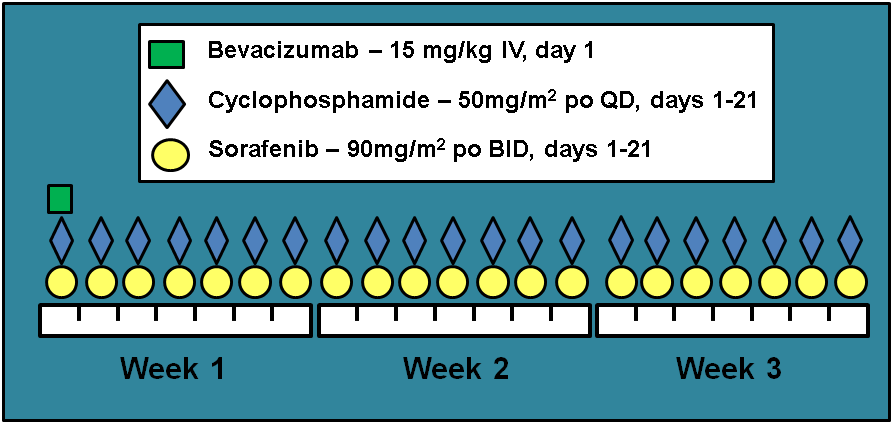


**Supplementary Figure 1.** Schema showing recommended phase 2 doses of regimen as per ANGIO1

## Supplementary Tables

| **Case** | **Description of Dosing Modifciation** |
| --- | --- |
| **1** | Bevacizumab held cycles 1-3, 27, 31-33 and 45 perioperatively, for wound healing and/or for proteinuria; Sorafanib dose reduced to 66% cycle 5 and further dose reduced to 33% cycle 7 for cytopenias and hand-foot syndrome (HFS); Sorafanib increased back to 66% dose reduction cycle 34 and to 50% cycle 35 following progressive disease |
| **2** | Cycle 5 delayed due bacteremia; Cyclophosphamide held cycle 8 for hematuria, resumed cycle 9 with mesna |
| **3** | Sorafanib decreased to daily cycle 4 for oral dysesthesia, intermittently increased to twice daily based on waxing and waning symptoms; Cyclophosphamide dose reduced 50% cycle 6 for thrombocytopenia and discontinued cycle 8; Cycle 7 delayed due to pancytopenia |
| **4** | Cycle 5 delayed due to elevated amylase/lipase; Sorafanib held on resumption of cycle 5, resumed cycle 7, held cycle 9 for recurrence of elevated amylase/lipase |
| **5** | Bevacizumab held cycles 1-3 for radiation therapy |
| **6** | Sorafanib held 3 days cycle 2 and resumed daily due to HFS, further decreased course 7; held cycle 5 for radiation therapy. Best Response at cycle 7, disease evaluation after cycle 3 with slight progression but continued therapy due to significant lapse in time between baseline evaluation and initiation of therapy. |
| **7** | Cyclophosphamide dose reduced to 66% cycle 2 due to pancytopenia, dosed every other day cycle 3, then held through cycle 7 then resumed; Sorafanib dose reduced cycle 4 to daily due to pancytopenia |
| **8** | Cyclophosphamide held cycle 5 due to neutropenia and thrombocytopenia; Sorafanib held cycle 8 due to pneumothorax, resumed 50% dose reduced, Bevacizumab held cycles 9 and 10 due to thrombocytopenia |
| **9** | Sorafanib dose reducted 33% cycle 4 due to HFS; Bevacizumab held cycle 8 perioperatively; Cycle 10 delayed due to radiation therapy |
| **10** | Sorafanib and Bevacizumab held cycle 3 due to wound healing, resumed cycle 4 with sorafanib dose reduced 50%; Sorafanib held multiple days cycle 5 for HFS |
| **11** | Sorafanib and Bevacizumab held cycle 4 due to wound healing, resumed cycle 5 with sorafanib dose reduced 50% |
| **12** | Cyclophosphamide held cycle 3 due to thrombocytopenia and neutropenia, resumed dose reduced 50% at cycle 5; Sorafanib held 1week cycle 7 for prolonged nausea and vomiting, dose reduced to daily on resumption |
| **13** | Treated locally; dosing modification data not available |
| **14** | No dosing adjustments |
| **15** | Treated locally; dosing modification data not available |
| **16** | Sorafanib dose reduced to daily cycle 3 due to HFS, resumed full dosing cycle 4 |
| **17** | Sorafanib held cycle 2 due to HFS, resumed at 50% dose reduction; Bevacizumab delayed in cycle 2 due to radiation therapy |
| **18** | No dosing adjustments |
| **19** | No dosing adjustments |
| **20** | No dosing adjustments |
| **21** | No dosing adjustments |
| **22** | Cycle 2 delayed due to HFS, Sorafanib dose reduced 50%; Cycle 9 delayed due to weight loss; Cycles 14 and 16 delayed due to thrombocytopenia; Cyclophosphamide delayed due to hemorragic cystitis and thrombocytopenia in cycles 14, 16 and 18, dose reduced 50% cycle 14 and discontinued due to thrombocytopenia cycle 19 |
| **23** | Cycle 4 delayed due to transaminitis; Cycle 8-11 with delays and intermittent holding chemotherapy; Cyclophosphamide dose reduced 50% with addition of mesna cycle 10; discontinuation of ANGIO1 following cycle 12 with 1 year interruption of therapy prior to progression; Cycle 16 held due to HFS; Intermittent holding of sorafanib and cyclophosphamide cycles 18, 28 and 29, with delay in cycle 29 due to hemorrhagic cystitis |
| **24** | Sorafanib dose reduced 50% cycle 1 due to HFS; Bevacizumab held cycle 2 and 3 for wound healing |
| **25** | Cycle 3 held due to admission for fever, neutropenia with bacteremia; Cycle 4 delayed due to admission, cyclophosphamide dose reduced 50% on resumption of chemotherapy |
| **26** | No dosing adjustments |
| **27** | Bevacizumab held cycle 3 for wound healing |
| **28** | Mesna included due to history of bladder manipulation |

**Supplementary Table 1.** Dose adjustments required according to cases pictorially shown in Figure 1
